# Effects of shift type, job position and a rigorous work period on physical- and performance-related attributes in female nursing workers

**DOI:** 10.1186/1550-2783-12-S1-P27

**Published:** 2015-09-21

**Authors:** Brennan J Thompson, Victoria K Banuelas, Chibuzo Akalonu, Matt S Stock, Dean Diersing

**Affiliations:** 1 Department of Health, Exercise and Sport Sciences, Muscular Assessment Laboratory, Texas Tech University, Lubbock, TX, 79409, USA

## Background

Healthcare workers exhibit among the highest injury rates of all occupations, with musculoskeletal injuries predominating. Anthropometric attributes and demanding work schedules may contribute to enhanced injury risks. Specifically, physical characteristics and performance capacities as well as accumulating fatigue from multiple work shifts may predispose workers to job-related injuries. Given the unique demands of day versus night shift work and varying job positions within the healthcare sector, it remains to be determined if these job characteristics may differentially impact nursing workers' physical and performance attributes. Therefore, the purpose of this study was to compare physical and performance attributes of both day and night shift nursing workers, and registered nurses (RNs) and nurses' aides (CNAs).

## Methods

Thirty-four full time female nurses (age = 32.9 ± 10.5 yrs, height = 163.4 ± 8.1 cm, mass = 73.8 ± 18.7 kg) working 12 h shifts participated in this study. Nurses were stratified by shift (day and night shift) and job position (RN and CNA). All groups were matched for age, and shift workers and job positions were matched across each category. Nurses visited the lab on three occasions with the first visit consisting of anthropometric assessments and familiarization on all performance tests. Visits two and three were within 24 h prior to, and 24 h following a four-day period that involved the nurses working three, 12 h shifts (36 h of work). Tests consisted of a computer-based psychomotor vigilance test (PVT) to assess reaction time and motivation, unipedal balance for 30 s, and isometric maximal strength (peak torque, PT; Nm) and rate of torque development (RTD, Nm·s^-1^) of the leg extensors and leg flexors. Subjects also completed a multidimensional fatigue questionnaire from which general and physical fatigue components were scored. Independent t-tests were used to assess differences between shifts and job positions.

## Results

CNAs (n = 10) were heavier compared to RNs (n = 24) (P = 0.05, 81.3 ± 21.0 and 68.8 ± 14.5 kgs, respectively), but no differences were shown for height (P = 0.28), nor for height or weight between night and day shift workers (p > 0.09). There were no differences for PVT, balance, leg extensors or flexors strength, or RTD variables for either shift or job position categories. There was however, a significant difference (P = 0.02) for work-induced change scores in leg flexors RTD with the night shift exhibiting greater declines (-46.2 ± 60.9 Nm·s^-1^) compared to the day shift (1.8 ± 58.7 Nm·s^-1^) workers. Also, CNAs exhibited greater (p < 0.03) general and physical fatigue scores compared to RNs (11.0 and 9.5 vs. 8.9 and 7.4, respectively) at baseline.

## Conclusion

These findings showed CNAs had greater body mass and subjective fatigue perception while night shift workers had greater work-induced declines in leg flexors explosive strength capacities. Thus, nutritional strategies may be tailored towards specific healthcare worker vulnerabilities, where weight management approaches may be pertinent for CNAs and improved explosive strength-endurance capacities for night shift workers. Also, nutritional aids designed for improving subjective mood and fatigue perception (i.e., stimulants, mood boosting nutritionals etc.) may be useful for enhancing recovery and reducing general and physical fatigue perception specifically in CNAs.

